# Medical Ethics Up-to-Date

**Published:** 1913-10

**Authors:** 


### OUR CONTEMPORARIES

### Medical Ethics Up-to-Date

*New Zealand Medical Journal,* June.

1. If called by night to attend a stranger at a distance, dress quickly and go, never stopping to ask who wants you, or if the bill will ever be paid, lest you be counted inhuman.

2. Never ask how many doctors are in attendance in a case, or how many kinds of patent medicines a patient is taking. Such curiosity on the part of the doctor is vulgar.

3. Never insult a stranger by asking for credentials, nor a patient by asking for money—pounds and shillings are the vernacular of bankers, lawyers, tradesmen, and "workers."

4. Never send in a bill; patients will think you are hard up, but pay *your* bills promptly. Send a check, it looks better.

5. In writing a prescription write illegibly. It does not matter. The druggist will put in "something just as good."

6. Be sure to mention the fact of your being overworked, and also cholecystitis, appendicectomy, opsonic index, operative work, toxæmia, words which impress the laity. Your wife must tell her friends how busy you are.

7. When going by a patient's home slip in socially and tell her of some interesting case, or some operation you have just performed, and incidentally mention how busy you are.

8. Never be friendly with any other doctor. It's unethical. If you think another doctor makes a guinea more a month than you do, cut him dead.

9. If another doctor's name is mentioned in your presence compress your lips, and the patient will understand that your hypertrophied good principles keep you from telling the truth, the whole truth, and a few other things. Do not have your principles so high you can't reach them.

10. If called in after another doctor has been treating a case of meningitis, make your diagnosis "inflammation of the brain," and be sure to say how much better it would have been had you been called in earlier.

11. It is understood that you would not interfere with gestation, but it is well to tell of the large sums of money you have been offered and refused.

12. If the other fellow does not think as you do, it proves his inferior intellect.

13. Jealousy and envy are the tributes paid to superiority.

14. Do not expect the "glad eye" when you give the "cold shoulder."

15. We have not enough "skin specialists" in the profession to offset the "wasters" in the laity.

16. Try not to have views. They are distressing—especially to others. If you must think, do it as quietly as possible.

17. Pretend that you are more skillful and proficient than others, and people will soon take you at your own estimation, especially if you can raise a small band of touts and claquers.

18. Endeavor to like each other, but if you can't—don't.

---

"Why Discriminate," says the Christian Science Sentinel, editorially, in its issue of August 16, referring to indictments against parents of victims of tuberculosis, etc., who have not had medical attendance. It quotes testimony in court showing cures of stomachs, kidney and heart trouble, consumption and various other ailments by Christian Science, after medical treatment had failed. It regards as unfair the fact that coroners ignore death occurring in patients of regular physicians. First just a word as to testimonials of cure. We are very skeptic of the ability of anyone, whether he knows anything about disease or not, either to diagnose, prognose or treat his own case. A grateful, though not discriminating patient, offered us a testimonial of cure the day before his sudden death, which had been foretold to his family. One of our old friends, a physician of ripe experience and sound judgment, died of a disease which he would have recognized clearly in a patient but of whose existence in his own case, he had not the slightest suspicion. Now, as to the unfairness of the discrimination. The law displays exactly the same unfairness in holding that the ordinary citizen may not kill and that the soldier, under certain circumstances, may; that the ordinary citizen cannot order another to stop, go ahead, turn to the right or left, or refrain from turning off a street when he wants to, but it gives this power to the policeman. Similar privileges are given to the plumber, the lawyer, many other persons each in a particular way and for a particular reason. The law cannot demand of any human being, superhuman ability, and it cannot, therefore, insist on success, merely on reasonable standards of preliminary qualification, good faith and attention to duty. Any Christian Scientist has the right to treat disease and to sign death certificates, if he will comply with the same requirements demanded of anyone else. There is no ultimate unfairness in this; it would be unfair to give to the Christian Scientist the privilege of a short cut to the prerogatives of the physician, the policeman, the soldier, the plumber, the pilot, the engineer and a host of others who undertake special responsibilities. *Why discriminate* against a sick child because his parents are Christian Scientists?

